# Fungi Treated with Small Chemicals Exhibit Increased Antimicrobial Activity against Facultative Bacterial and Yeast Pathogens

**DOI:** 10.1155/2014/540292

**Published:** 2014-07-09

**Authors:** Christoph Zutz, Dragana Bandian, Bernhard Neumayer, Franz Speringer, Markus Gorfer, Martin Wagner, Joseph Strauss, Kathrin Rychli

**Affiliations:** ^1^Institute for Milk Hygiene, University of Veterinary Medicine Vienna, Veterinaerplatz 1, 1210 Vienna, Austria; ^2^AIT-Austrian Institute of Technology GmbH, University and Research Campus Tulln, Konrad Lorenz Straße 24, 3430 Tulln on the Danube, Austria; ^3^Fungal Genetics and Genomics Unit, Department of Applied Genetics and Cell Biology, BOKU University of Natural Resources and Life Sciences Vienna, Konrad Lorenz Straße 24, 3430 Tulln on the Danube, Austria

## Abstract

For decades, fungi have been the main source for the discovery of novel antimicrobial drugs. Recent sequencing efforts revealed a still high number of so far unknown “cryptic” secondary metabolites. The production of these metabolites is presumably epigenetically silenced under standard laboratory conditions. In this study, we investigated the effect of six small mass chemicals, of which some are known to act as epigenetic modulators, on the production of antimicrobial compounds in 54 spore forming fungi. The antimicrobial effect of fungal samples was tested against clinically facultative pathogens and multiresistant clinical isolates. In total, 30 samples of treated fungi belonging to six different genera reduced significantly growth of different test organisms compared to the untreated fungal sample (growth log reduction 0.3–4.3). For instance, the pellet of *Penicillium restrictum* grown in the presence of butyrate revealed significant higher antimicrobial activity against *Staphylococcus* (*S.*) *aureus* and multiresistant *S. aureus* strains and displayed no cytotoxicity against human cells, thus making it an ideal candidate for antimicrobial compound discovery. Our study shows that every presumable fungus, even well described fungi, has the potential to produce novel antimicrobial compounds and that our approach is capable of rapidly filling the pipeline for yet undiscovered antimicrobial substances.

## 1. Introduction

For decades, one of the main sources for the discovery of novel antimicrobial drugs has been the screening of fungal cultures for bioactive natural compounds [1, 2]. However, due to the increasing risk of rediscovery of antimicrobial compounds, many screening programs have been stopped in the last decade. Nevertheless, the increasing rate of appearance of resistant pathogens, the decreasing lifetime of antibiotics in clinical use, and the decline of novel antibiotic candidates in the pipeline raise the necessity to develop new strategies for antimicrobial compound discovery [3]. In fungi, antimicrobial compounds are mainly produced during the secondary metabolism (SM). Ongoing genome sequencing of fungi leads to new insights into the quantity of genes related to the SM [4, 5]. There is evidence that the number of genes involved in the SM is higher than the number of characterized fungal secondary metabolites, thus indicating a high number of so far unknown compounds [4, 6]. Genes involved in the SM of fungi are often organized in gene clusters, which are mainly in a transcriptional silenced state, if fungi are grown under standard laboratory culture conditions [6]. These silenced gene clusters hold the potential to code for proteins involved in the biosynthesis of new antimicrobial compounds [7]. Several approaches to activate such “cryptic” gene clusters have been performed, for example, by* trans*-over-expressing cluster-resident transcription factor [8], by deleting precursor-consuming pathways [9], by cocultivation with bacteria [10], and by interference with the epigenetic silencing pathway [11]. The silencing of gene clusters has been linked to epigenetic mechanisms which influence the formation of “facultative heterochromatin” [12]. Two of the major epigenetic signals of chromatin regulation are acetylation of histones, which results mainly in activation of gene transcription, and methylation of histones and DNA, which is mainly linked to silencing of transcription [13]. Recent studies showed that various low molecular mass chemicals, in this study named small chemicals (SCs), are able to inhibit histone deacetylases (HDACs) and DNA methyltransferases (DNMTs) in fungi [14]. Furthermore, it was demonstrated in fungi that specific SCs influence the secondary metabolite profile [15, 16].

The aim of this study was to investigate the effect of different SCs on the production of antimicrobial compounds in 54 spore forming fungi. We used six SCs, of which three were HDAC inhibitors: valproic acid (VPA), which inhibits class I HDACs and induces proteosomal degradation of class II HDACs [17, 18]; the antifungal compound trichostatin A (TSA), which inhibits class I and II HDAC activity [19]; and sodium butyrate (butyrate), which induces differentiation in eukaryotic cell lines via HDAC inhibition [20–22]. In addition, we used the DNA methylation inhibitor 5-azacytidine (AZA), which incorporates into RNA and to less extend into DNA, thus leading to elevated levels of hypomethylated DNA [23, 24]. Furthermore, we included N-acetyl-D-glucosamine (GlcNAc), which induces secondary metabolite production on* Streptomyces* spp. [25] and nitric oxide (NO) a phytohormone, which has been shown to regulate gene expression in fungi and might therefore play a role in SM regulation [26, 27].

## 2. Materials and Methods

### 2.1. Bacterial and Fungal Strains Used in This Study

Antimicrobial activity of fungal cultures was tested against the Gram positive and Gram negative facultative pathogens* Staphylococcus* (*S.*)* aureus* (Rosenbach 1884, ATCC6538) and* Pseudomonas* (*P.*)* aeruginosa* (Migula 1900, ATCC9027) and the yeast pathogens* Candida* (*C.*)* albicans* (Robin Berkhout, ATCC10231); all are obtained from DSMZ, Germany. For detailed analysis in an enhanced screen, we used* Klebsiella pneumonia* ATCC13883,* Escherichia coli* MC1061, the yeast-like potential pathogenic fungus* Exophiala* (*E.*)* dermatitidi*s (internal strain collection number 132), and three multiresistant clinical isolates: extended-spectrum betalactamase (ESBL)* Klebsiella* (*K*.)* pneumonia* (B100173), ESBL* Escherichia* (*E*.)* coli* (B300129), and methicillin-resistant* S. aureus* (MRSA) B337919 (kindly provided by Analyse Biolab GmbH, Austria). Initial cultivation was performed on tryptone soya agar (Oxoid, USA) for 18 h at 37°C. One single colony was inoculated in tryptone soya broth supplemented with 6 g/L yeast extract (TSBY, Oxoid, USA) and incubated at 37°C for 6 h for* S. aureus* and* P. aeruginosa* and for 10 h for* C. albicans*. All fungal strains used in this study were obtained from the fungal strain collection of the AIT (Austrian Institute of Technology, Fungal Genetics and Genomics Unit, Table 1). Identification of the fungi was performed according to Klaubauf et al. [28].

### 2.2. Small Chemicals

SC (all Sigma Aldrich, USA) stock solutions were prepared as recently described [16]. Shortly, valproic acid (VPA, 2-propylpentanoic acid) was dissolved in 96% ethanol to a final concentration of 60 *μ*M. Trichostatin A (TSA, 7-4-(dimethylamino)phenyl-N-hydroxy-4,6-dimethyl-7-oxohepta-2,4-dienamide, 0.5 *μ*M), sodium butyrate (butyrate, 9 *μ*M), 5′-azacytidine (AZA, 4-amino-1-*β*-D-ribofuranosyl-1,3,5-triazin-2(1H)-1, 2 *μ*M), and N-acetyl-D-glucosamine (GlcNAc, 0.5 *μ*M) were dissolved in PBS (pH 7). To prepare a stock solution that releases nitric oxide, a stock solution of NaNO_2_ (0.3 M in PBS) was mixed at a ratio 1 to 1 with acidified Moser medium (pH 5.0). Stock solutions were stored at −20°C for not more than 6 months.

### 2.3. Preparation of Fungal Cultures

The fungal strains used in this study were thawed from cryogenic storage vials, streaked on malt extract agar plates (MEA; Oxoid, USA), and grown at room temperature (RT) until sporulation took place. To harvest the spores, the agar plate was drenched with PBS supplemented with 0.1% tween and scraped with a sterile cotton stick. Purity of spore solution was assessed (Nikon, Eclipse E200 40x magnification) and spores were incubated a second time on MEA plates. After incubation at RT for 10 (8 and 20) days, the spores were harvested and inoculated at a concentration of 10^6^ spores/mL in 20 mL minimal fungal medium (Moser medium, composed of 10 g/L glucose, 0.2 g/L yeast extract, 2 g/L tryptic-digested peptone from casein, 0.5 g/L KH_2_PO_4_, 50 mg/L inositol, 75 mg/L CaCl_2_, 10 mg/L FeCl_3_, 150 mg/L MgSO_4_, and 10 mg/L MnSO_4_).

The fungal strains were grown in absence and presence of the different SCs (final concentration of 5 *μ*M, except 1.5 mM for NO) at RT for 72 h in the absence of light on a rotary shaker at 180 rpm.

Of each fungal culture, four different fractions were prepared: supernatant, extracted supernatant, pellet, and extracted pellet. The supernatant and mycelium were harvested using a sterile cellulose filter. The supernatant was furthermore filtered a second time through a cellulose filter and an aliquot was directly used in the antimicrobial activity screen (supernatant).

To prepare the extracted supernatant, the remaining supernatant was extracted with acetone 1 : 1 (v/v) and agitated for 1 h at room temperature followed by extraction with chloroform 1 : 1 (v/v) and agitated for 30 min. The extract was incubated at 4°C until complete separation of the organic and the water phase took place. The chloroform phase was transferred into a new tube, dried under reduced pressure in a centrifugal vacuum concentrator (Mivac, Fisher Scientific, USA), and resuspended in 1 mL Moser medium supplemented with 10% DMSO (Sigma Aldrich, USA).

The fungal mycelium was dried between two sheets of Whatman filter paper (VWR, Germany). For the preparation of the pellet extract, 200 mg of the mycelium was mixed with 1 mL PBS and 1 g of glass beads of 2 different diameters (*∅* 0.75 and 1 mm, Fisher Scientific, USA). Fungal cells were lysed mechanically using a Fast Prep FP120 (three times for 30 sec) and centrifugated two times at 5500 rpm for 10 min at 4°C and 800 *μ*L of the supernatant was filtered through a syringe filter (Rotilabo Syringe filter *∅* 0.22 *μ*m; Roth, Germany). For the preparation of the extracted pellet, 1 g of the mycelium was mixed with 1 mL of chloroform and glass beads according to the manufacturer's recommendation. The cells were lysed as described above and 600 *μ*L of the supernatant was transferred into a new tube. The supernatant was further evaporated in a centrifugal vacuum concentrator at 30°C and resuspended in Moser medium supplemented with 10% DMSO.

### 2.4. Antimicrobial Activity Screen

For the setup of the initial antimicrobial activity screen, we investigated the growth of* S. aureus, C. albicans, and P. aeruginosa* at 37°C for 48 h in four cultivation media: Luria-Bertani (LB, Oxoid, USA), tryptone soya broth supplemented with yeast extract (TSBY), Winge (20 g/L glucose, 3 g/L yeast extract), and yeast extracted peptone dextrose (YEPD, Oxoid, USA) using glass tubes and 96-well microtiter plates. TBSY was chosen as cultivation medium. After three cultivations for 18 h in TSBY at 37°C, cultures were diluted to an OD_600_ of 0.05 for* S. aureus* and of 0.5 for* C. albicans* and* P. aeruginosa*. The initial screen was performed in 96-well flat bottom microtiter plates (Greiner bio-one, Austria). Per well, 80 *μ*L of fresh TSBY was mixed with 100 *μ*L of fungal extract (supernatant, extracted supernatant, pellet, and extracted pellet) and additionally 20 *μ*L of the test organism culture (OD_600_ of 0.05 for* S. aureus*, 0.5 for* P. aeruginosa,* and* C. albicans*) was added to reach the final reaction volume of 200 *μ*L. As controls, Moser medium alone, with the specific SCs with 10% DMSO, was used. The growth was measured after 0, 6, and 18 h of incubation at 37°C using a multiplate photometric reader (Tecan Infinite 200pro, Switzerland). Every experiment was performed in three biological replicates in triplicate. To validate the screening method, the intra- and interassay coefficients of variation (CV) were calculated according to Reed et al. [29].

For the enhanced antimicrobial activity screen,* E. dermatitidis* was cultivated three times for three days at 37°C, diluted to an OD_600_ of 0.5, and incubated in TSBY in glass tubes for 54 h at 37°C. OD_600_ measurements were performed at 0, 6, 18, 36, and 54 h due to the slow growth rate. In addition,* Klebsiella pneumonia*,* E. coli,* ESBL* K*.* pneumonia*, ESBL* E*.* coli,* and the MRSA strain were cultivated for 18 hours in TSBY at 37°C. Growth experiments were performed with a start inoculum of 0.05 in glass tubes at a final volume of 2 mL and OD_600_ was measured in a spectrophotometer (UV1800, Shimadzu, Japan).

### 2.5. Evaluation of Antimicrobial Activity of Fungal Cultures

The growth of the test organisms in the presence of the SC treated fungal samples was compared to the growth in the presence of the untreated fungal samples and to the growth of the test organisms alone. In addition, growth in the presence of the different SCs and DMSO was used as a control.

Of all fungal samples displaying antimicrobial activity, a colony forming unit assay (CFU) was performed. The determination of CFU was performed simultaneously with the OD_600_ measurements by serial dilution plating on tryptone soya agar plates, and the CFUs were evaluated 24 h after incubation at 37°C. The CFU assay was done in three independent biological replicates performed in triplicate. Controls were used similar to the OD_600_ measurement.

### 2.6. Cytotoxicity Assay

A cytotoxicity assay measuring lactate dehydrogenase (LDH) release was performed using human intestinal epithelial (Caco2) and human hepatocytic (HepG2) cells. The cells were cultivated in Eagle's minimum essential medium (MEM) containing 2 mM L-glutamine, 10% fetal bovine serum (FBS), 100 units/mL penicillin, 100 mg/mL streptomycin sulfate, 0.25 mg/mL amphotericin B, and 1% nonessential amino acids (NEAA; all PAA) at 37°C in a humidified atmosphere (95% relative humidity) containing 5% CO_2_.

The cells were seeded in 96-well plates (5 × 10^4^ per well) and incubated until a confluent cell layer developed. For the cytotoxicity assay, cells were treated with 50 *μ*L fungal fraction and 50 *μ*L MEM for 24 h at 37°C and LDH leakage was determined using the LDH* in vitro* toxicology assay kit according to the manufactures' instructions (Sigma-Aldrich, USA). The percentage of dead cells was calculated using a standard curve of serial diluted lysed cells (100% dead cells). Each experiment was performed three times in triplicate.

### 2.7. Statistical Analysis

Microsoft Excel 2007 and Sigma plot 11.0 (Sysat Software Inc., Great Britain) were used for statistical analysis. Values were compared statistically using *t*-test and Dunnett test (two independent variables). *P* values <0.05 were considered to be significant.

## 3. Results

### 3.1. Development of the Antimicrobial Activity Screen

For the setup of the antimicrobial activity screen, we tested four liquid media (LB, TSBY, Winge, and YEPD) commonly used in microbiology (see Table S1 in Supplementary Material available online at http://dx.doi.org/10.1155/2014/540292). TSBY showed the smallest difference in growth dynamics and best growth rate for all three test organisms in both the glass tube and 96-well microtiter plate assay (Table S1). In addition, we tested different inoculum sizes (OD_600_ 0.001–0.1) and revealed the lowest differences in growth dynamics using an inoculum size of OD_600_ 0.005 for* S. aureus* and of OD_600_ 0.05 for* P. aeruginosa* and* C. albicans*. Additional, by using these inoculum sizes, all three test organisms were in a comparable growth phase after 6 and 18 h of incubation at 37°C, after 6 h in the mid- or end logarithmic, and after 18 h in the stationary growth phase (Figure S1). In parallel, CFUs were determined after 6 and 18 h to confirm the growth dynamics (data not shown). For the antimicrobial activity screen, the test organisms were grown at a ratio 1 to 1 in TSBY and the fungal samples were resuspended in Moser medium with and without DMSO. Moser medium did not influence the growth dynamics of all three test organisms. Furthermore, neither one of the SCs at the given concentration nor 2.5% DMSO had an effect on the growth of all three test organisms (data not shown).

### 3.2. Validation of the Antimicrobial Activity Screen

To validate the variability and reproducibility of the bioactivity screens, we calculated the intra- and interassay coefficients of variation (CV). The intra-assay CV describes the reproducibility of technical replicates; interassay CV specifies the reproducibility of biological replicates [29]. Overall, the intra-assay CV is lower than the interassay CV (Table 2). The variability of growth of both assays is below 10% for* S. aureus* and* P. aeruginosa* at 6 and 18 h and for* C. albicans* at 6 h. The highest inter- and intra-assay CV was determined after 18 h for* C. albicans*; therefore, growth of* C. albicans* was only evaluated at the time point of 6 h, respectively.

### 3.3. Influence of the Maturation of Spores on the Antimicrobial Activity

Visible sporulation of all fungi took place after 8 days of incubation at RT. We observed that the age of the spores used for the fungal cultures influenced the production of antimicrobial compounds. For instance, the untreated supernatant of* Oidiodendron* (*O.*)* cerealis* (NG_p39) showed a significantly higher antimicrobial activity against* S. aureus*, if we inoculated 10- and 20-day-old spores, whereas no antimicrobial activity could be observed by using 8-day-old spores (Figure S2). Since no difference was observed between spores grown for either 10 or 20 days, we decided to harvest all spores of all fungi after 10 days of incubation.

### 3.4. SCs Induce Antimicrobial Activity in Fungi

In total, we investigated antimicrobial activity of fungal samples of 54 strains belonging to 11 different genera, which were grown in the presence of six different SCs. To include both intra- and extracellular antimicrobial compounds and the whole range of polar and apolar compounds, we prepared four fractions of each fungal culture: the supernatant and the mycelium, both crude and extracted. In total, we determined the effect of 1512 different fungal fractions on the growth of three different test organisms.

Thirty fungal samples of treated fungi belonging to six different genera reduced significantly growth of the test organism compared to the untreated fungal sample (Table 3). Of these 30 samples, only two, namely, the supernatant of* A. clavatus* (L19) and* H. koningii* (NG_14 BRE-1P1), showed already antimicrobial activity in the untreated sample, although to a lower extent compared to the treated fraction.

All six SCs induced the production of antimicrobial compounds at least in the fraction of one fungus; additional increased bioactivity was observed in all four different fungal fractions, respectively. In total, 12 fungal samples showed activity against* C. albicans*, 10 against* S. aureus,* and 8 against* P. aeruginosa*. Overall, we observed cell count reductions varying between 0.3 and 4.3 (Table 3).

**Figure 1:**
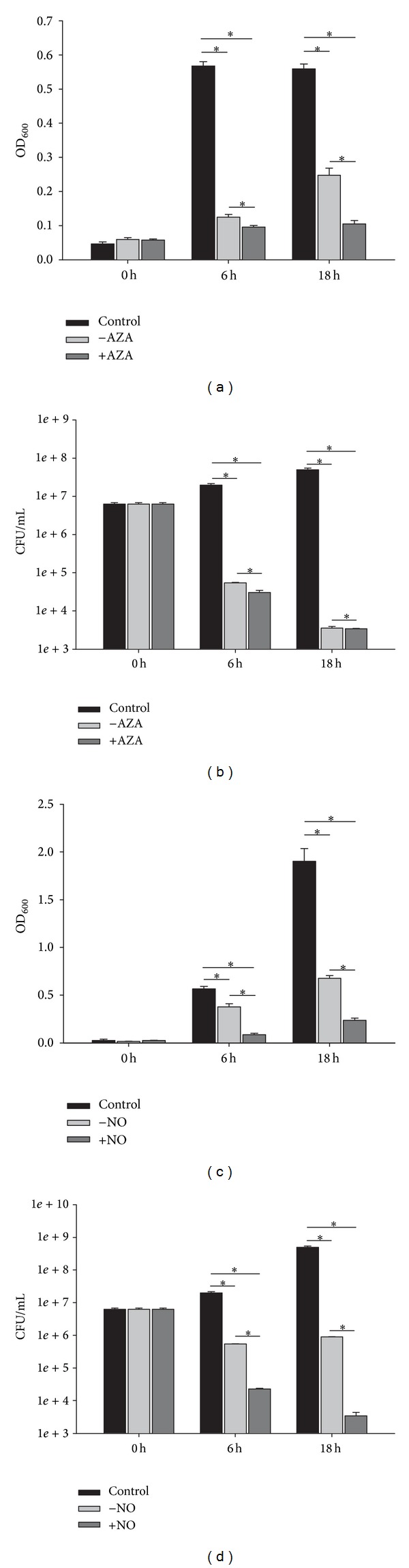
Antimicrobial effects of the AZA treated (+AZA) and untreated (−AZA) supernatant of* A. clavatus* (L19) on the growth of* S. aureus* ((a): OD_600_, (b): CFU/mL). Antimicrobial effects of the NO treated (+NO) and untreated (−NO) supernatant of* H. koningii* (NG_14 BRE-1P1) on the growth of* P. aeruginosa* ((c): OD_600_, (d): CFU/mL). Control comprises* S.aureus* or* P. aeruginosa* grown without fungal sample. Data is presented as mean values ± standard deviations of three biological replicates performed in triplicate. ∗indicates significant difference (*P* < 0.05).

The most salient growth suppressing effects were as follows: we detected a strong antimicrobial activity against all three test organisms in the supernatant of* A. clavatus* (L19). Although antimicrobial activity was already detected in the supernatant of the untreated fungus, treatment with VPA, TSA, butyrate, AZA, and GlcNAc resulted in a significantly increased bioactivity. The highest antimicrobial activity, however, was observed in the supernatant of* A. clavatus* (L19) grown in the presence of AZA, resulting in a reduction of* S. aureus* numbers of 4.3 log CFU (Figures 1(a) and 1(b)). In addition, these supernatants inhibited also growth of* E. dermatitidis, Klebsiella pneumonia*,* E. coli,* ESBL* K*.* pneumonia*, ESBL* E*.* coli,* and MRSA strain (data not shown).

Increased antimicrobial activity against* P. aeruginosa*, resulting in a CFU log reduction of 4, was measured also in the supernatant of* H. koningii* (NG_14 BRE-1P1) grown in the presence of NO compared to untreated supernatant (Figures 1(c) and 1(d)). However, in this fungus, bioactivity was already detected in the supernatant of the untreated fungi.

**Figure 2:**
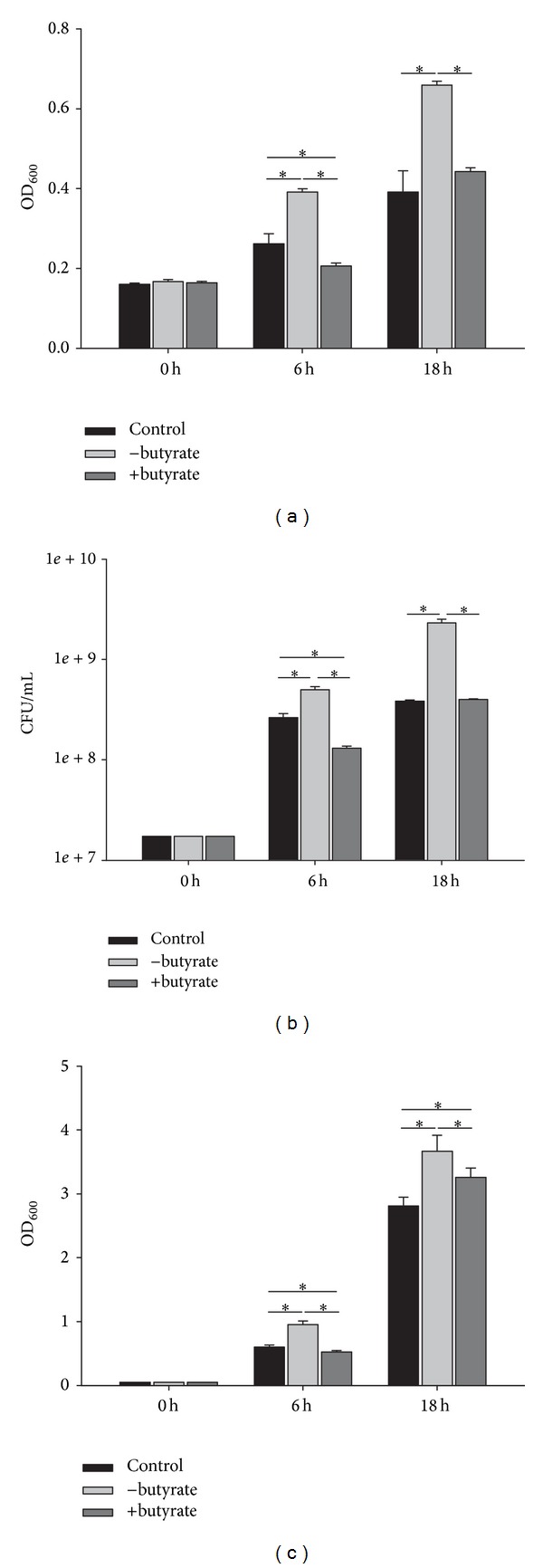
Antimicrobial activity of the pellet of* P. restrictum* (PRF-18) grown in the absence (−butyrate) and presence (+butyrate) of butyrate against* S. aureus* ((a): OD_600_, (b): CFU/mL) and the MRSA strain B337919 ((c): OD_600_). Control comprises* S. aureus* or MRSA grown without fungal samples. Data is represented as mean values ± standard deviations of three biological replicates performed in triplicate. ∗indicates significant difference (*P* < 0.05).

The pellet of* P. restrictum* (PRF-18) grown in the presence of butyrate showed antimicrobial activity against* S. aureus* compared to the untreated fungal sample (and the control), even though the growth of the test organisms was increased in the presence of the untreated pellet (Figure 2(a)). In parallel, this fungal fraction revealed a 1.4 log reduction in* S. aureus* numbers (Figure 2(b)). In the enhanced screen, this* P. restrictum* (PRF-18) sample showed antimicrobial activity against the MRSA strain (Figure 2(c)).

**Figure 3:**
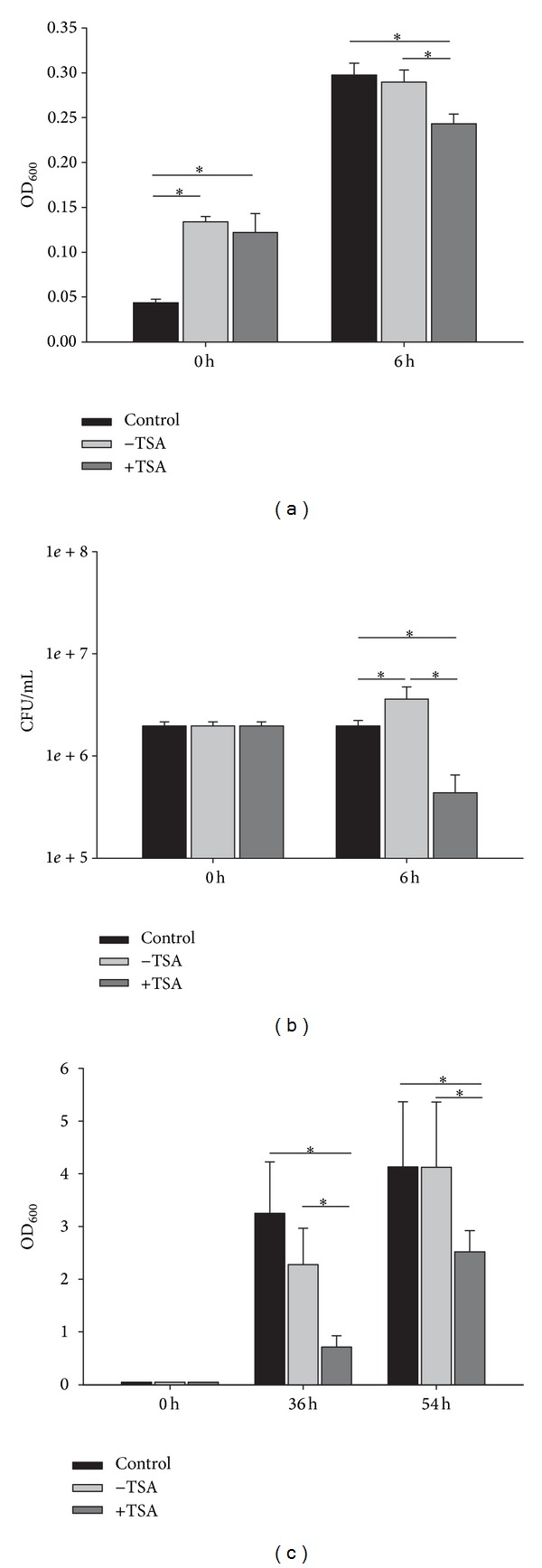
Antimicrobial activity against* C. albicans* ((a): OD_600_, (b): CFU/mL) and* E. dermatitidis* ((c): OD_600_) in the extracted pellet of* P. crustosum* (D_D27) grown with (+TSA) and without TSA (−TSA). Controls comprise* C. albicans* and* E. dermatitidis* grown without fungal sample. Data comprises mean values ± standard deviations of three biological replicates performed in triplicate. ∗indicates significant difference (*P* < 0.05).

With regard to* P. crustosum* (D_D27), TSA increased the antimicrobial activity against* C. albicans* in the extracted pellet (Figure 3(a)). In parallel, the number of viable* C. albicans* cells decreased, (CFU log reduction of 1.1, Figure 3(b)) and the antimicrobial effect of this fungal sample on the growth of* E. dermatitidis*, a slow growing fungus, was even stronger compared to the effect on* C. albicans* (Figure 3(c)).

**Figure 4:**
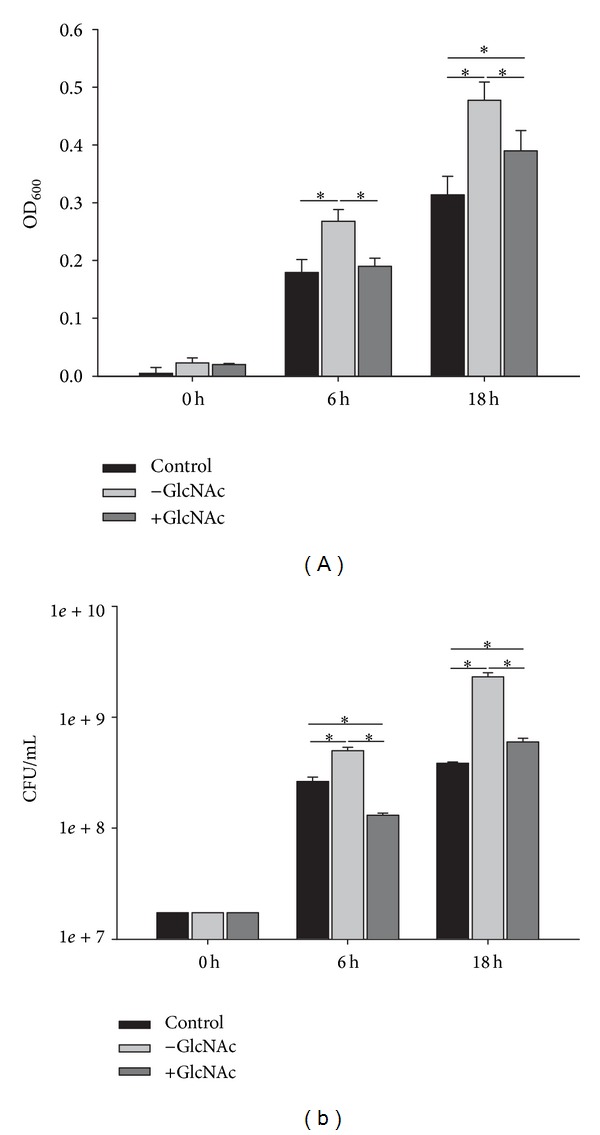
Effect of the GlcNAc treated (+GlcNAc) and untreated (−GlcNAc) supernatant of* P. spinulosum* (Li0102XIX) on the growth of* S. aureus* measured by OD_600_ (a) and CFU/mL (b). Control comprises* S. aureus* grown without fungal sample. Data are represented as mean values ± standard deviations of three biological replicates performed in triplicate. ∗indicates significant differences (*P* < 0.05).

*P. spinulosum* (Li0102XIX) was analytically challenging, since the supernatant displayed weak turbidity leading to higher OD_600_ at the start point (0 h). The presence of GlcNAc increased antimicrobial activity against* S. aureus* in the supernatant of this organism (Figure 4). We observed that the untreated crude fungal supernatant already increased the growth* S. aureus* compared to the control.

### 3.5. Cytotoxicity of Fungal Samples

**Figure 5:**
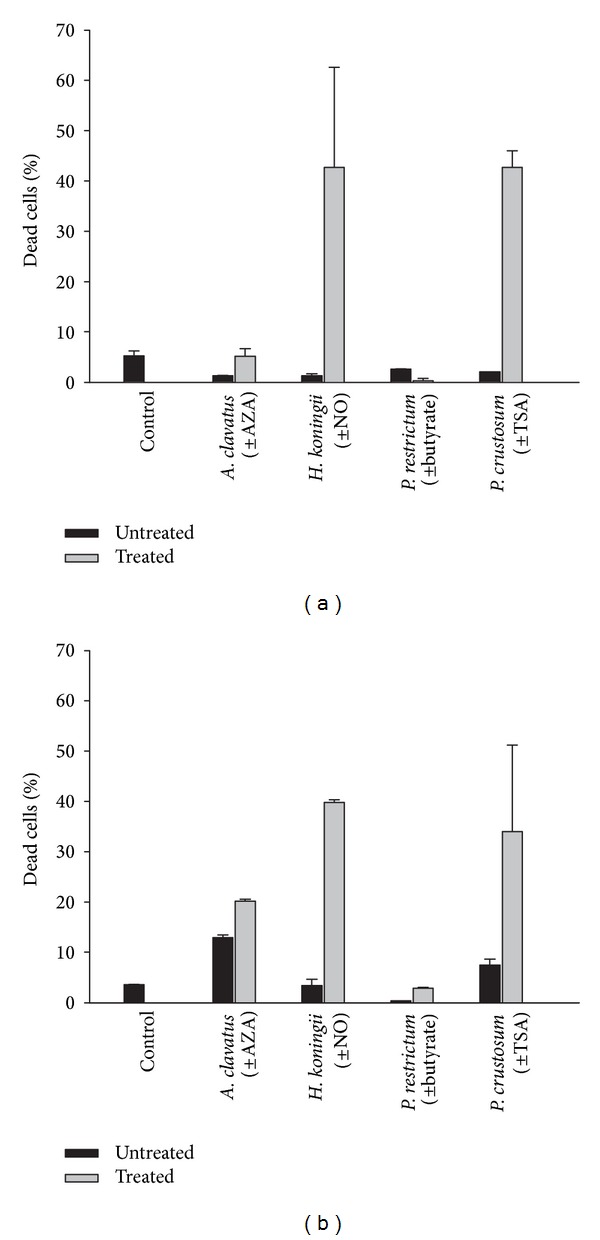
Cytotoxic effects of the supernatant of* A. clavatus* (L19), the supernatant of* H. koningii* (NG_14 BRE-1P1), the pellet of* P. restrictum* (PRF-18), and the extracted pellet of* P. crustosum* (D_D27) incubated with (treated) and without (untreated) the respective SC using human intestinal epithelial Caco2 (a) and hepatocytic HepG2 (b) cells. Control comprises Moser medium. Values, given as % of dead cells, represent mean values ± standard deviations of three biological replicates performed in triplicate.

To get a first insight into the host susceptibility, we investigated the cytotoxicity of the respective fungal fraction of* A. clavatus* (L19),* H. koningii* (NG_14 BRE-1P1),* P. restrictum* (PRF-18), and* P. crustosum* (D_D27) using two human cell lines (Caco2 and HepG2, Figure 5). The cytotoxic effects of the fungal samples were strain and cell line dependent (% of dead cells: 0–42%).

The incubation of the cells with Moser medium alone resulted in 3.6 to 5% dead cells. All but one tested fractions of fungi treated with the respective SC showed higher cytotoxicity compared to the untreated crude fraction. Only the pellet of* P. restrictum* (PRF-18) grown in the presence of butyrate revealed fewer dead Caco2 cells. Overall, both fractions of* P. restrictum* (PRF-18) were not cytotoxic, revealing 0–2.8% dead cells after 24 h of incubation, whereas the treated fungal fractions of* H. koningii* (NG_14 BRE-1P1) and* P. crustosum* (D_D27) were highly cytotoxic, resulting in 40% dead cells. The treated fraction of* A. clavatus* (L19) displayed moderate cytotoxicity ranging from 8% dead cells for Caco2 to 21% for HepG2 cells.

## 4. Discussion

The increasing need for novel antimicrobial drugs raises the necessity to establish alternative sources and high-throughput methods for antimicrobial compound discovery. Recent progress in fungal sequencing and in the understanding of epigenetic regulation mechanisms suggests a large number of so far unknown secondary metabolites which may include antimicrobial active compounds [4, 6]. In this study, we describe and evaluate a strategy to discover new natural antimicrobial compounds combining a yet unexploited approach using small mass chemicals to modify the secondary metabolite production in fungi with a sensitive antimicrobial activity screen.

The presented screening method was designed to determine the growth of three test organisms, which are known to acquire antibiotic resistance. These species are therefore commonly used to assess antimicrobial activity. The growth medium TSBY, an unselective growth medium, is suited for receiving low variability with respect to growth dynamics. Therefore, biased positive results due to nutrient limitation, selective pressure, or medium variability are negligible. When validating the antimicrobial screens, the variability and reproducibility revealed remarkable low inter- and intra-assay CVs, which indicates low media batch variation and an excellent opportunity to detect also weak antimicrobial activity. Both CVs were below 10% for all test organisms after 6 and 18 h of growth. Comparable studies using screening systems for bacterial susceptibility against common antibiotics reported inter-assay CV values of 10–20% depending on the test organism [30]. The only exception was the growth variability of* C. albicans* when incubated for 18 h.* C. albicans* clusters at the bottom of the reaction well resulting in a poorer resuspension before OD measurements.

In total, 54 fungi including 12* Aspergilli* and 30* Penicillia* strains were treated with six SCs and screened for novel antimicrobial compounds in four fractions. All fungi were grown in the same medium and kept under constant conditions to avoid influence of biotic and abiotic factors on the expression of secondary metabolites. The high number of* Aspergilli* and* Penicillia* strains was chosen due to the large amount of genomic, proteomic, and ecological data available for these genera. In addition, recent studies showed that even closely related fungi share only a small number of similar secondary metabolites and that horizontal gene transfer of biosynthesis clusters of secondary metabolites is a scarce event [31, 32].

We detected 30 samples of treated fungi belonging to six different genera, with seven* Penicillia* strains among them, which reduced significantly growth of the test organism compared to the untreated fungal samples. All SCs used in this study effected the production of antimicrobial compounds at least in one fungus. However, whether the effect of the SC on the production of antimicrobial compounds is due to epigenetic modulation in the specific fungus must still be investigated. Interestingly, except for* A. clavatus*, increased antimicrobial activity was only detected in one of the four fungal fractions of all fungi. Furthermore, growth was inhibited either of yeast, gram positive, or gram negative bacteria (except* A. clavatus*). This is in line with recent findings that SCs are target specific, inducing specific secondary metabolite production [16, 33].

Excluding* A. clavatus,* seven of these modified fungal fractions showed activity against yeast, five against gram positive bacteria, and only three against gram negative bacteria. The small number of fungal fractions displaying activity against gram negative bacteria underlines the poor availability of antibiotics against gram negative bacteria and underlines that few novel candidates are in the pipeline [34].

In the supernatant of untreated and treated* A. clavatus,* we revealed high antimicrobial activity against all tested organisms and cytotoxicity.* A. clavatus* is known to produce the mycotoxin patulin, which is cytotoxic and highly bioactive also against gram positive and negative bacteria [35, 36]. Both the antimicrobial activity and cytotoxicity were increased if this fungus was grown in the presence of all SCs. These findings are in line with one of our studies, which shows that the SCs increased, among other mycotoxins, patulin production in* A. clavatus* [16].

In addition, the crude supernatant of* H. koningii* revealed already high antimicrobial activity against* P. aeruginosa*; however, the presence of NO resulted in increased bioactivity and cytotoxicity. It has been described that* H. koningii* produces various koninginins, which display a wide range of bioactivities including antimicrobial activity against gram negative bacteria [37, 38]. Our observation that also the addition of NO increases the antimicrobial activity of* H. koningii* (and also in* P. miczynskii*) supports the hypothesis that the phytohormone NO influences SM production also in fungi, not only in plants [26, 27, 39]. At present, it is unknown if NO influences chromatin structure in fungi; therefore, further research is needed to study the role of NO in the regulation of secondary metabolite production.

The finding that specific SCs increase the production of specific secondary metabolites like patulin and presumably koninginins highlights that small mass chemicals potentially inducing epigenetic modulation could be used for biotechnological approaches to increase the yield of specific compounds. Since* A. clavatus* and* H. koningii* showed antimicrobial activity already in the crude supernatant without any SCs, we disclosed these fractions from further analysis, because of the allegedly risk of rediscovery of known compounds.

Apart from* A. clavatus* and* H. koningii*, CFU log reductions between 0.3 and 1.4 were observed in the fungal samples, which corresponds to a relatively weak antimicrobial activity compared to the bacteriocidal effects of commercially available antimicrobial compounds. Optimization of biotic and abiotic factors, for example, temperature, light, pH, nutrients, and oxygen availability, which are known to influence production of SM [40], could increase the yield of the antimicrobial compounds. Furthermore, there are indications that the age of the spores influences the production of antimicrobial compounds [41]. This is in line with our finding that, for example, in* O. cerealis*, which is known to produce the antimicrobial active compounds fuscin and clerocidin [42], antimicrobial bioactivity was only observed if the spores were grown for at least 10 days. Therefore, optimization of the age of spores could also increase the yield of antimicrobial compounds.

The observation that the pellet of* P. restrictum* grown in the presence of butyrate is not cytotoxic and revealed antimicrobial activity against* S. aureus* (CFU log reduction of 1.4) and MRSA indicates a high potential for the discovery of a novel antimicrobial compound in this fungal sample. No antimicrobial compounds active against gram positive bacteria have yet been described in any* P. restrictum* strain. It is known that* P. restrictum* produces calbistrin, which inhibits growth of fungi [43]. However, no antimicrobial activity against* C. albicans* has been observed in the fraction of the tested* P. restrictum* strain. Whether butyrate acts as an epigenetic modulator generally in fungi (including* P. restrictum*) is still unknown. So far, the epigenetic effect of butyrate via inhibition of HDACs (mainly of class II and III) has only been described in mammalian cells [22].

We observed that growth of* S. aureus* was increased in the presence of several fungal samples, both crude and treated, which indicates that fungal metabolites in both the pellet and the supernatant can be used by* S. aureus* as an additional nutrient source. In two of these cases (supernatant of GlcNac treated* P. spinulosum* and pellet of* P. restrictum* treated with butyrate), the presence of the SC treated fraction reduced significantly growth compared to the untreated fraction and also to the control, which suggests the presence of antimicrobial compounds. However, we cannot fully exclude that the specific SC also downregulates metabolites, which are used by* S. aureus* as nutrients. Taken together, the high number of antimicrobial active fungal samples in this study underlines the potential of this approach, combining treatment of fungi with small chemicals with a sensitive screening tool. In a common first screen of a set of 100 000–200 000 samples, 0.1–1% are estimated to be initial bioactive hits [44]. Up to 90% of these hits are predicted to be discarded because their bioactivity is a result of either a combination of weak bioactivities, due to biological variations or a dereplication of known compounds. This results in approximately 10–200 samples which are taken to further isolation and identification [44]. Our study comprising 1350 samples of 54 fungi detected 14 fungal samples containing potential novel antimicrobial compounds, which will be isolated and characterized in future studies. Compared to the estimated gain of 0.01–0.1% of an industrial screen, our method reached a gain of approximately 0.7%. This indicates a large number of so far unknown antimicrobial compounds in already screened and characterized fungi which are accessible through to potential epigenetic regulation.

## 5. Conclusion

In conclusion, our study is the first to show the potential of SCs to induce the production of “cryptic” antimicrobial compounds against gram positive, gram negative bacteria, and yeasts. The diversity of the antimicrobial hits in the initial screen indicates that every presumable fungus, even well described fungi, has the potential to produce novel antimicrobial compounds that warrant discovery. In general, epigenetic modification of fungi could reduce the risk of rediscovery of known compounds. Furthermore, the used method combining treatment of fungi with small chemicals with a sensitive screening tool can easily be upscaled for high-throughput screening programs, discovering novel antimicrobial natural products.

## Supplementary Material

Table S1: Different growth dynamics of *S. aureus, P. aeruginosa* and *C. albicans* using four growth media (LB, TSBY, Winge and YEPD). The experimantes were performed in glass tubes at 37°C.
Figure S1: Growth of *S. aureus* inoculum size OD_600_ 0.005), *P. aeruginosa* (inoculum size OD_600_ 0.05) and *C. albicans* (inoculum size OD_600_ 0.05) at 37°C in 96-well plates using TSBY.
Figure S2 shows the influence of spore maturation on the production of antimicrobial compounds in *O. cerealis*.

## Figures and Tables

**Table 1:** Fungal strains used in this study.

Species	Internal strain code	Isolation source	Country	Sequencing°
*Aspergillus clavatus *	L19	Air sample	Austria	ITS-LSU
*Aspergillus flavipes *	NG_p18	Grassland soil	Austria; Riederberg	ITS
*Aspergillus flavus *	L16	Air sample	Austria	ITS-LSU
*Aspergillus niger *	D_An	Indoor air	Austria	ITS-LSU
*Aspergillus nomius *	E06			ITS-LSU
*Aspergillus reptans *	D_D37	Indoor air	Austria	ITS-LSU
*Aspergillus sclerotiorum (section circumdati) *	L07	Air sample	Austria	ITS-LSU
*Aspergillus spp. *	RJW		Austria; Vienna	ITS
*Aspergillus sydowii *	EVU-01	Indoor wall	Austria; Vienna	ITS
*Aspergillus terreus *	ÖFI 05-01B			ITS
*Aspergillus versicolor *	MB0839-7	Indoor air car	Austria	ITS-LSU
*Aspergillus westerdijkiae *	D_At	Indoor air	Austria	ITS-LSU
*Cladosporium sphaerospermum *	D_D48	Indoor air	Austria	ITS-LSU
*Clavicipitaceae spp. *	NG_p36	Agricultural soil		ITS
*Hypocrea koningii *	NG_14 BRE-1P1	Agricultural soil	Austria; Maissau	ITS
*Hypocrea lixii *	NG_p16	Agricultural soil	Austria; Niederschleinz	ITS
*Isaria farinosa *	KF0909_H8	Indoor material sample	Austria	ITS-LSU
*Lecythophora hoffmannii *	NG_p46	Agricultural soil	Austria; Maissau	ITS
*Oidiodendron cerealis *	NG_p39	Agricultural soil	Austria; Maissau	ITS
*Penicillium alberechii *	Hbs-K14	Air sample	Austria; Vienna	ITS
*Penicillium biourgeianum *	GAbP	Indoor air	Germany	ITS
*Penicillium brevicompactum *	Hbs-K13	Air sample	Austria; Vienna	ITS
*Penicillium canescens *	NG_p02	Grassland soil	Austria; Riederberg	ITS
*Penicillium corylophilum *	D_D42	Indoor air	Austria	ITS-LSU
*Penicillium chrysogenum *	MC-A12	Silica plates air condition	Austria; Vienna	ITS
*Penicillium commune *	MCB11	Silica plates air condition	Austria; Vienna	ITS
*Penicillium crustosum *	D_D27	Indoor air	Austria	ITS-LSU
*Penicillium decumbens *	D_D54	Food sample	Austria	ITS-LSU
*Penicillium decaturense *	RSF-Q205	Root sample Quercus	Austria; Redlschlag	ITS
*Penicillium echinulatum *	W121	Warburgia ugandensis	Uganda	ITS-LSU
*Penicillium funiculosum *	NG_p14	Agricultural soil		ITS
*Penicillium glabra *	D_D28	Indoor air	Austria	ITS-LSU
*Penicillium glabrum/thomii *	Li0102II	Spruce needle	Austria; Kalwang	ITS-LSU
*Penicillium glandicola *	NG_24	Agricultural soil	Austria; Maissau	ITS
*Penicillium islandicum *	NG_P43	Agricultural soil	Austria; Riederberg	ITS
*Penicillium islandicum/rugulosum *	NG_P23	Agricultural soil		ITS
*Penicillium janthinellum *	NG_23	Agricultural soil	Austria; Purkersdorf	ITS
*Penicillium lividum *	Li0102XII	Spruce needle	Austria; Kalwang	ITS-LSU
*Penicillium miczynskii *	A04	Air sample	Austria	ITS-LSU
*Penicillium olsonii *	D_D38	Indoor air	Austria	ITS-LSU
*Penicillium piceum *	D_D04	Indoor air	Austria	ITS-LSU
*Penicillium pinophilum/verruculosum *	MX-C1	biocontrol product	Mexico	ITS
*Penicillium polonicum *	D_D47	Indoor air	Austria	ITS-LSU
*Penicillium restrictum *	PRF-18	Ectomycorrhizal Salix root tip	Austria; Arnoldstein	ITS-LSU
*Penicillium roseopurpureum-related *	D_D20	Indoor air	Austria	ITS-LSU
*Penicillium rugulosum *	ÖFI 05-01F		Austria; Vienna	ITS
*Penicillium soppi *	RSF-Q201	Root sample Quercus	Austria; Redlschlag	ITS
*Penicillium spinulosum *	Li0102XIX	Spruce needle	Austria; Kalwang	ITS-LSU
*Penicillium ochrochloron *	NG_25	Grassland soil	Austria; Riederberg	ITS
*Phanerochaete chrysosporium *	P.c.			ITS
*Rhizopus oryzae *	R.R.	Rapeseed pellets		ITS
*Trichoderma atroviride *	GAbT	Indoor air	Germany	ITS
*Trichoderma tomentosum *	NG_02	Agricultural soil	Austria; Niederschleinz	ITS
*Trichoderma viride *	NG_p24	Grassland soil	Austria; Riederberg	ITS

°Identification of the fungi was based on sequencing of the fungal internal transcribed spacer (ITS) region and partial LSU regions (ITS/LSU) according to Klaubauf et al. [28].

**Table 2:** Intra- and inter-assay coefficient of variation (CV) of biological and technical replicates.

	Inter-assay CV [%]		Intra-assay CV [%]	
	6 h	18 h	6 h	18 h
*S. aureus *	3.1	5.8	2.1	2.9
*P. aeruginosa *	4.9	6.5	2.0	4.3
*C. albicans *	9.4	24.4	7.6	17.4

**Table 3:** Fungal samples showing increased antimicrobial activity after SC treatment compared to the untreated fungal sample.

Fungal strain	Fungal fraction	SC	Target	Log reduction°
*Aspergillus clavatus*∗	Supernatant	AZA	*S. aureus *	4.28 ± 0.745
		Butyrate		3.01 ± 0.521
		TSA		3.89 ± 0.637
		VPA		3.75 ± 0.419
		GlcNAc		0.72 ± 0.187
		AZA	*P. aeruginosa *	4.03 ± 0.923
		Butyrate		3.24 ± 0.427
		TSA		4.12 ± 0.845
		VPA		4.04 ± 0.736
		GlcNAc		1.25 ± 0.249
		AZA	*C. albicans *	2.01 ± 0.423
		Butyrate		1.84 ± 0.359
		TSA		2.04 ± 0.476
		VPA		3.12 ± 0.863
		GlcNAc		1.36 ± 0.419

*Clavicipitaceae spp. *	Extracted supernatant	Butyrate	*C. albicans *	1.10 ± 0.301

*Hypocrea koningii*∗	Supernatant	NO	*P. aeruginosa *	4.00 ± 0.673

*Isaria farinosa *	Extracted supernatant	Butyrate	*C. albicans *	0.37 ± 0.084

*Penicillium alberechii *	Supernatant	TSA	*S. aureus *	0.47 ± 0.035
		Butyrate		0.51 ± 0.074
		VPA		0.49 ± 0.063

*Penicillium crustosum *	Extracted pellet	TSA	*C. albicans *	1.10 ± 0.451

*Penicillium decaturense *	Supernatant	AZA	*C. albicans *	0.35 ± 0.032
		Butyrate		0.30 ± 0.012
		TSA		0.31 ± 0.057

*Penicillium miczynskii *	Extracted supernatant	NO	*C. albicans *	0.74 ± 0.087

*Penicillium restrictum *	Pellet	Butyrate	*S. aureus *	1.40 ± 0.204

*Penicillium soppi *	Extracted supernatant	TSA	*P. aeruginosa *	0.56 ± 0.096

*Penicillium spinulosum *	Supernatant	GlcNAc	*S. aureus *	0.94 ± 0.061

*Trichoderma atroviride *	Pellet	AZA	*P. aeruginosa *	0.42 ± 0.085

°CFU log reduction comparing treated with untreated fungal fractions determined after 6 hours of growth at 37°C. Data are represented as mean values ± standard deviations of three biological replicates preformed in triplicate. ∗Untreated fungal fraction showed already antimicrobial activity.
