# Supplementary material for: Fungi Treated with Small Chemicals Exhibit Increased Antimicrobial Activity against Facultative Bacterial and Yeast Pathogens
Source: Biomed Res Int. 2014 Jul 9;2014:540292. doi: 10.1155/2014/540292 (PMC4119895; doi:10.1155/2014/540292)
Supplement: Supplementary file 1 — Table S1: Different growth dynamics of S. aureus, P. aeruginosa and C. albicans using four growth media (LB, TSBY, Winge and YEPD). The experimantes were performed in glass tubes at 37°C. Figure S1: Growth of S. aureus inoculum size OD600 0.005), P. aeruginosa (inoculum size OD600 0.05) and C. albicans (inoculum size OD600 0.05) at 37°C in 96-well plates using TSBY. Figure S2 shows the influence of spore maturation on the production of antimicrobial compounds in O. cerealis. [file 540292.f1.pdf]

Epigenetically modified fungi exhibit increased antimicrobial activity against facultative bacterial and yeast pathogens.

Christoph Zutz, Dragana Bandian, Bernhard Neumayer, Franz Speringer, Markus Gorfer, Martin Wagner, Joseph Strauss, Kathrin Rychli

Supplementary Material

Table S1 Growth of *S. aureus*, *P. aeruginosa* and *C. albicans* obtained in LB, TSBY, Winge and YEPD media incubated in glass tubes at 37°C.

	S.aureus		
	8h	24h	48h
LB	+++	+++	+++
TSBY	+++	+++	+++
Winge	+	+	+
YEPD	+	++	++

	P.aeruginosa		
	8h	24h	48h
LB	++	++	++
TSBY	++	++	+++
Winge	+	+	++
YEPD	+	++	++

	C.albicans		
	8h	24h	48h
LB	+	++	++
TSBY	+	+++	+++
Winge	++	++	+++
YEPD	+	++	+++

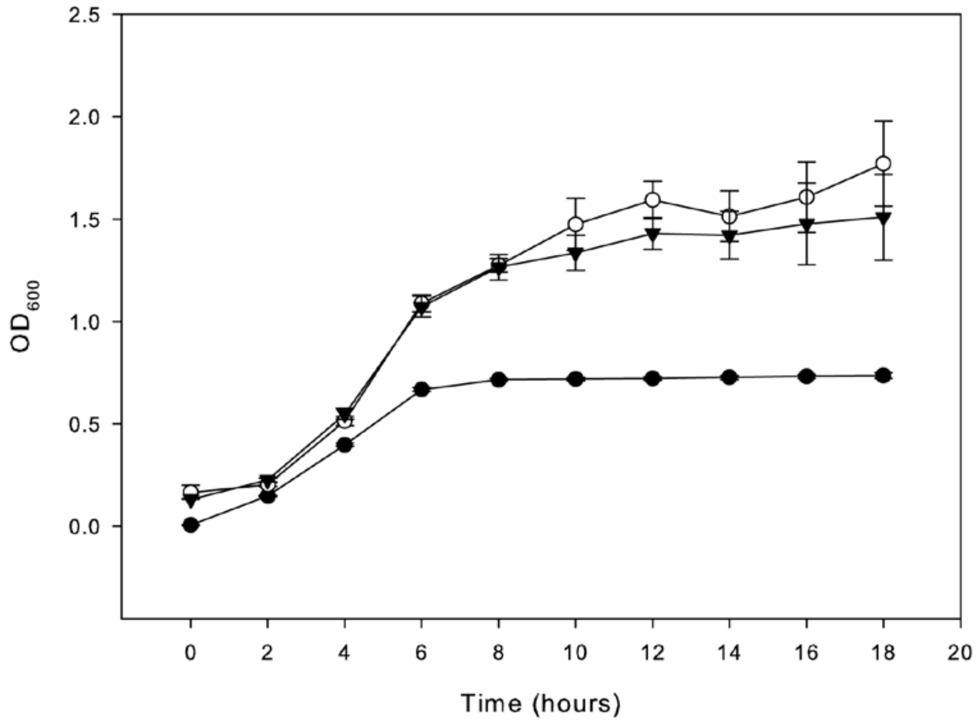

Figure S1. Growth of *S. aureus* (●, inoculum size OD_{600} 0.005), *P. aeruginosa* (○, inoculum size OD_{600} 0.05) and *C. albicans* (▼, inoculum size OD_{600} 0.05) at 37°C in 96-well plates using TSBY. Mean values ± standard deviations of three independent replications are indicated.

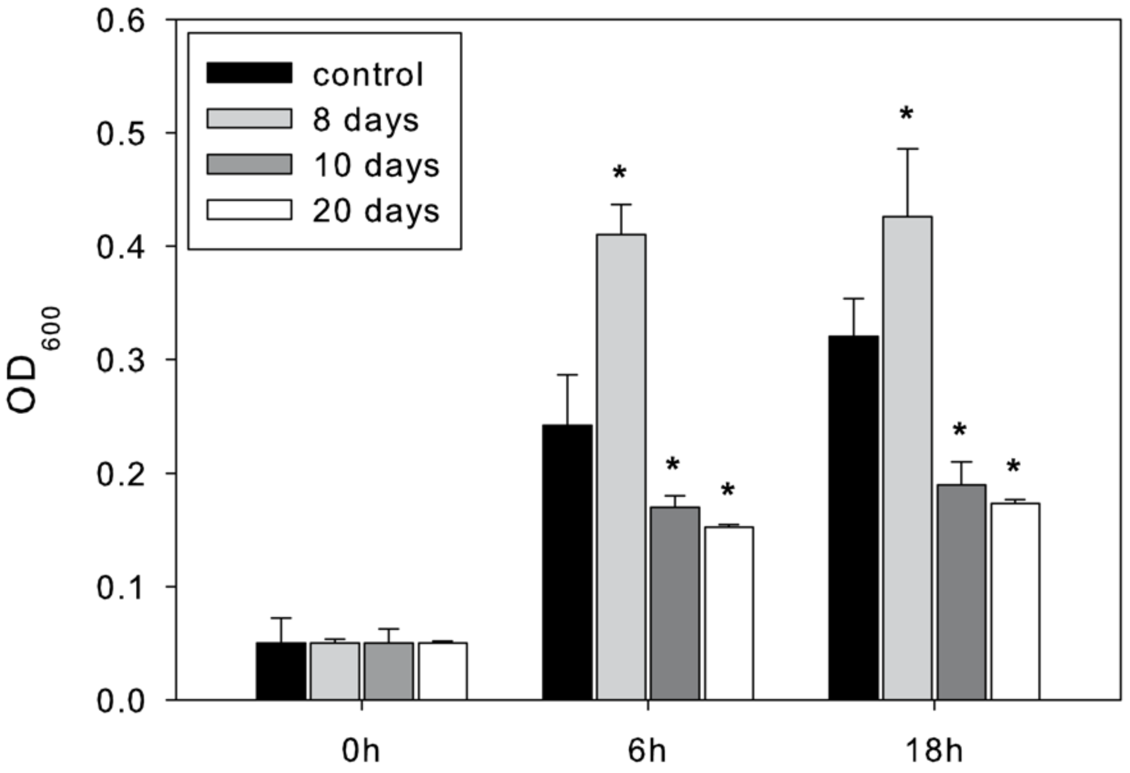

Figure S2. Influence of the spore maturation (8-, 10- and 20-day-old spores) on the antimicrobial activity in the supernatant of *O. cerealis* (NG_p39) against *S. aureus*. Values are mean values ± standard deviations of three independent biological replicates, *indicates significant difference compared to control ($P<0.05$).
